# Assessing Higher-Order Visual Processing in Cerebral Visual Impairment Using Naturalistic Virtual-Reality-Based Visual Search Tasks

**DOI:** 10.3390/children9081114

**Published:** 2022-07-26

**Authors:** Claire E. Manley, Christopher R. Bennett, Lotfi B. Merabet

**Affiliations:** The Laboratory for Visual Neuroplasticity, Department of Ophthalmology, Massachusetts Eye and Ear, Harvard Medical School, Boston, MA 02114, USA; cemanley@meei.harvard.edu (C.E.M.); christopher.richard.bennett@gmail.com (C.R.B.)

**Keywords:** cerebral visual impairment (CVI), visual search, eye tracking, virtual reality, higher order visual processing, attention, visual perception

## Abstract

Cerebral visual impairment (CVI) is a brain-based disorder associated with the maldevelopment of central visual pathways. Individuals with CVI often report difficulties with daily visual search tasks such as finding a favorite toy or familiar person in cluttered and crowded scenes. We developed two novel virtual reality (VR)-based visual search tasks combined with eye tracking to objectively assess higher order processing abilities in CVI. The first (virtual toybox) simulates a static object search, while the second (virtual hallway) represents a dynamic human search task. Participants were instructed to search for a preselected target while task demand was manipulated with respect to the presence of surrounding distractors. We found that CVI participants (when compared to age-matched controls) showed an overall impairment with visual search on both tasks and with respect to all gaze metrics. Furthermore, CVI participants showed a trend of worsening performance with increasing task demand. Finally, search performance was also impaired in CVI participants with normal/near normal visual acuity, suggesting that reduced stimulus visibility alone does not account for these observations. This novel approach may have important clinical utility in helping to assess environmental factors related to functional visual processing difficulties observed in CVI.

## 1. Introduction

Clinical ophthalmic testing typically relies on standard measures of visual function, such as visual acuity, contrast sensitivity, and perimetry. In the setting of ocular-based impairment, these measurements can be helpful in characterizing the profile of an individual’s visual deficits and informing appropriate adaptive strategies (e.g., increased magnification for reduced visual acuity) [1]. However, in the case of a brain-based impairment, such as cerebral (or cortical) visual impairment (CVI), the profile of observed deficits is often very complex and heterogeneous, making the connection between measures of visual function and appropriate adaptive strategies more difficult to surmise [2]. 

CVI has been defined as “verifiable visual dysfunction associated with damage to retrochiasmatic pathways and cerebral structures that cannot be attributed to disorders of the anterior visual pathways or potentially co-occurring ocular pathology” [3]. Common causes of CVI include hypoxic-ischemic injury, trauma, infection, as well as genetic and metabolic disorders [4,5]. Early neurological injury is believed to lead to the maldevelopment or malfunction of key visual processing structures and pathways [6]. Individuals with CVI often present with lower-order visual deficits such, as reduced visual acuity and contrast sensitivity, as well as impaired visual field sensitivity and ocular motor functions (e.g., fixation, saccades, and pursuit movements) [4,7]. However, assessing visual functions alone fails to capture the complex clinical profile observed in this population. Indeed, higher-order perceptual processing impairments related to visual identification, visuospatial processing, and attention are also prominent features of CVI [4,7,8,9]. This is evident even in the case where an individual has normal/near normal visual acuity [2]. Finally, these associated dysfunctions can have a profound impact on the development and functional independence of an individual [8,10,11].

Individuals with CVI often report challenges with searching and extracting visual information from complex (i.e., cluttered or crowded) scenes [12,13,14,15]. These perceptual difficulties can make an individual feel overwhelmed and anxious or, in some cases, appear inattentive. For example, they may easily identify a favorite toy when presented in isolation, yet have difficulties finding it when placed in a box filled with other toys [11]. They may also struggle to identify a familiar person (including a family member) in a very busy crowd [13,14,15]. 

To provide a more comprehensive evaluation of CVI, it is therefore crucial to assess not only standard measures of visual function but also higher-order processing abilities in relation to functional vision. In other words, it is important to characterize how an individual uses their vision in real-world situations [16,17]. Failing to do so can lead to an underestimation of visual difficulties or worse, a dismissal of reported complaints and even misdiagnosis [18,19,20,21]. Furthermore, developing novel and adaptive assessments are particularly relevant in the case of individuals with CVI who may not be able to verbalize the nature of their visual difficulties, have challenges undergoing formal ophthalmic testing, or have perceptual deficits despite having visual functions within normal or near normal range. 

Visual perceptual abilities can be assessed in a variety of ways including neuropsychological testing (e.g., Developmental Test for Visual Perception). However, these tests are typically not carried out as part of a standard ophthalmological exam [2,22]. Furthermore, there are a number of factors to consider that may potentially bias testing results. These include the need for verbal and/or manual motor responses, sufficient visual acuity to view fine-detailed images, and an overall comprehension of task requirements. Finally, while standardized neurophysiological testing can provide important information regarding developmental progress, it nonetheless remains difficult to disentangle the nature of observed perceptual impairments and, ultimately, how testing results translate to real-world tasks and situations. Thus, there remains a pressing need to develop objective assessment tools that can characterize higher-order visual perceptual functional vision abilities in the context of common and everyday activities and capture the complex and heterogeneous profile of individuals with CVI [2,20,23].

To address these gaps, our group has been developing desktop virtual reality (VR)-based visual search tasks to simulate the exploration of a naturalistic scene. Realistic VR environments can be created so that they are easy and intuitive to interact with and have high behavioral relevance and participant engagement [16,24]. Furthermore, environments can be designed to objectively test the effect of manipulating various factors in relation to scene complexity and task demands [16]. In this direction, search behavior can serve as a proxy to assess higher-order perceptual processing and functional vision [25]. Visual search studies have provided important insights regarding processing abilities in relation to visual identification, visuospatial processing, and the deployment of attention [26]. Thus, by combining carefully designed VR environments with objective eye tracking and multiple measures of performance, this approach avoids many potential testing confounds, such as the necessity for verbal/manual responses or low participant engagement. At the same time, the effect of manipulating environmental factors on performance can be explored and adaptive strategies can be potentially identified. 

In a set of preliminary studies, we found that children and adolescents with CVI showed clear impairments with respect in visual search performance when interacting with naturalistic VR-based environments [27,28]. Building on these early findings, we now provide further characterization of higher-order perceptual processing abilities in CVI and quantify the effect of varying task demands with respect to scene complexity. The first task (called “the virtual toybox”) represents a static object search, while the second (referred to as “the virtual hallway”) corresponds to a dynamic human search task. In both environments, visual search performance in relation to scene complexity (e.g., clutter and crowding) was objectively assessed by manipulating the presence and number of surrounding distractor items. As a secondary assessment, we also investigated the effect of visual acuity on performance by dichotomizing our CVI study participants into two groups based on having normal/near normal (20/15 to 20/25 Snellen) or reduced (20/30 to 20/100 Snellen) acuity. 

Consistent with clinical accounts [12,13,14,15], we hypothesized that CVI participants would show an overall impairment in search performance as indexed by multiple behavioral outcome measures (including success rate and reaction time) as compared to age-matched controls with neurotypical development. Further, we hypothesized that as a group, individuals with CVI would show greater impairment with increasing task demands associated with scene complexity. Finally, we surmised that search performance in CVI would be impaired even in the setting of normal/near normal visual acuity. This would be consistent with the maldevelopment or malfunction of key processing structures and pathways implicated in higher-order visual processing. 

## 2. Methods 

### 2.1. Study Participants

A total of 32 individuals with neurotypical development aged between 11 and 23 years old (mean age 20.31 years ± 6.03 SD; 10 males) and 25 participants with CVI aged between 8 and 27 years old (mean age 17.48 years ± 5.21 SD; 11 males) were enrolled in the study. Twenty-seven controls and twenty-four CVI subjects participated in the toybox task, and twenty-five controls and twenty-four CVI subjects participated in the hallway task. Thus, of the total 57 participants, 75% (*n* = 24) controls and 88% (n = 22) CVI individuals, participated in both experimental tasks. Across both experiments, comparing CVI subjects and controls revealed no statistically significant difference with respect to age (t(38.17) = 1.82, *p* = 0.08, d = 0.49). Written informed consent was obtained from all participants and a parent/legal guardian (in the case of a minor) prior to commencing the study. The study was approved by the Investigative Review Board at the Massachusetts Eye and Ear in Boston, MA, USA, and carried out in accordance with the Code of Ethics of the World Medical Association (Declaration of Helsinki) for experiments involving humans.

Comparative controls had normal or corrected to normal visual acuity and no previous history of any ophthalmic (e.g., strabismus, amblyopia) or neurodevelopmental (e.g., attention deficit hyperactivity disorder) conditions. Participants with CVI were all previously diagnosed by experienced clinicians specializing in neuro-ophthalmic pediatric care. Diagnosis was based on a directed and objective assessment of visual functions (e.g., visual acuity, contrast, visual field perimetry, color, and ocular motor functions), thorough refractive and ocular examination, as well as extensive and integrated review of medical (including developmental, birth, and gestational) history, neuroimaging, and electrophysiology records [4,29,30]. Further input regarding visual behaviors were collected from available questionnaires and inventories [31,32] for the purposes of formalizing the diagnosis [18]. All participants with CVI had visual impairments related to pre- or perinatal neurological injury and/or neurodevelopmental disorders. Causes of CVI included hypoxic-ischemic injury related to prematurity (including periventricular leukomalacia; PVL), hypoxic/ischemic encephalopathy (HIE), seizure disorder, as well as genetic and metabolic disorders. Associated neurodevelopmental comorbidities included spastic and dystonic cerebral palsy. Best-corrected visual acuities in the better seeing eye ranged from 20/15 to 20/100 Snellen (0.07 to −0.12 logMAR equivalent). All participants had visual acuities, intact visual field function within the area corresponding to the visual stimulus presentation, as well as fixation and binocular ocular motor function sufficient for the purposes of completing the task requirements (including eye-tracking calibration). Exclusion criteria included any evidence of oculomotor apraxia, intraocular pathology (other than mild optic atrophy), uncorrected strabismus, visual field deficit corresponding to the area of testing, uncontrolled seizure activity, as well as cognitive deficits precluding the participant from understanding the requirements of the study. 

### 2.2. Focus Group Study

To inform the initial design of the VR-based environments and tasks, we carried out a focus group study to elicit input from potential clinical participants, parents, teachers of the visually impaired, and clinicians. Using a series of questionnaires and iterative testing (quantified using Likert scales and open-ended questions), two real-world tasks were identified as particularly challenging for individuals with CVI (see [27,28] for further details regarding questionnaire design). Specifically, these were (1) identifying a favorite toy in a cluttered environment and (2) identifying and following a familiar person walking in a crowd. Follow-up questionnaires revealed that the presence of visual clutter and crowd size were potentially important factors that could influence task performance. 

### 2.3. Behavioral Tasks and Visual Stimulus Design

The two corresponding environments (referred to as the virtual toybox and virtual hallway) were developed using the Unity 3D game engine version 5.6 (Unity Technologies, San Francisco, CA, USA) running on an Alienware Aurora R6 desktop computer (Intel i5 processor, NVidia GTX 1060 graphics card, and 32 GB of RAM; Alienware Corporation). Three-dimensional object models were created using Blender modeling software (Blender Foundation), and 3D human models were created in Adobe Fuse CC and rigged for animation in Adobe Mixamo (Adobe Systems Inc., San Jose, CA, USA). 

For both experimental tasks, participants were seated comfortably in a quiet room, in front of a desktop 27” LED monitor (either a ViewSonic Widescreen: 60Hz, 1920 × 1080 resolution or BenQ: 144Hz, 1920 × 1080 resolution), and with the eye tracker unit (see below) mounted on the lower portion of the monitor. At a viewing distance of 50–60 cm, the virtual toybox subtended approximately 32 to 38 × 32 to 38 deg of visual angle, while the virtual hallway subtended 58 to 68 × 32 to 38 deg of visual angle. Participants were reminded to maintain their gaze on the screen during testing but otherwise were able to move their head freely.

Visual search patterns (corresponding to X and Y coordinate positions of gaze on the screen) were captured using a Tobii 4C Eye Tracker system (90 Hz sampling frequency, Tobii Technology AB, Stockholm, Sweden). Prior to each experiment, eye-tracking calibration was performed on each participant (Tobii Eye Tracking Software, v 2.9 calibration protocol), which took less than one minute to complete. The process included a 7-point calibration task (screen positions: top-left, top-center, top-right, bottom-left, bottom-center, bottom-right, and center-center) followed by a 9-point post-calibration verification (i.e., the same 7 calibration points plus center-left and center-right positions). Accuracy criterion was determined by gaze fixation falling within a 2.25 arc deg radius around each of the 9 points and was further confirmed by visual inspection prior to commencing data collection.

#### 2.3.1. Experimental Task 1: The Virtual Toybox

**Figure 1:**
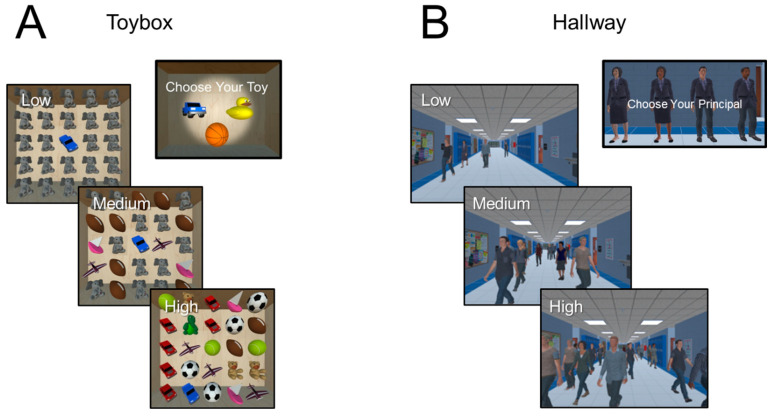
Experimental Design. (**A**) The virtual toybox (static object) visual search task. Prior to data collection, participants were asked to select a target toy from 3 possibilities (a blue truck, yellow duck, or orange basketball; right panel). The selected toy (in this case, a blue truck) was presented in a random location among distractor toys within a 5 × 5 array. Task demand associated with image clutter was manipulated at 3 levels (low, medium, high) corresponding to the number of unique distractor toys in the array (note the total number of the toys were constant across all levels of difficulty). (**B**) The virtual hallway (dynamic human) visual search task. Participants selected a target (the principal of a fictitious school) from 4 possibilities (balanced by gender and race; right panel). The selected principal (in this case, a Caucasian female) enters the hallway scene from either side and walks toward the observer. Task demand associated with crowd density was manipulated at 3 levels (low, medium, high) corresponding to the number of individuals walking in the hallway.

The virtual toybox was designed as a static object search task representing a simulated rendering of a box with a 5 × 5 array of static toys shown in canonical view and without overlap (Figure 1A). The task was presented in a trial-by-trial fashion and viewed from an overhead, first-person perspective to simulate the appearance of looking down into the box. Prior to commencing the study, participants were instructed to select a target toy presented in isolation and rotating around the y axis (options were a yellow duck, orange basketball, and blue truck). This was done to confirm that the participant could identify the search target in isolation as well as promote engagement and enhance the immersive feel of the task. Once selected, participants were instructed to search, locate, and fixate the specific target toy placed in a random location among a heterogeneous array of surrounding distractor toys. The target toy remained constant and was completely unique in terms of shape and features in order to create a “pop out” effect when presented with the other distractor toys (akin to a feature search). The size of the toys and spacing between them remained constant throughout all trials.

The primary factor manipulated was the number of unique toys in the grid serving as surrounding distractors. Three distractor levels, classified as low (1 to 3 unique toys), medium (4 to 6), and high (7 to 9), were equally presented on each trial and in pseudorandom order. Note that on each trial, the number of presented objects was fixed (i.e., a constant array or “set size” of 25 objects), and thus, only the location of the target and number of unique surrounding distractors were varied. A trial consisted of 4 s viewing the toybox scene followed by a 2 s blank grey screen with a central fixation target. Each run consisted of 35 trials (lasting approximately 3.5 min), and participants completed a total of 3 runs (i.e., 3 × 35 trial blocks for a total of 105 trials) with a brief rest period between. Total testing time was approximately 15 to 20 min for each participant (for further details regarding task design and preliminary results, see [27]). 

#### 2.3.2. Experimental Task 2: The Virtual Hallway

The virtual hallway was designed as a dynamic human search task and represented a simulated rendering of a hallway of a fictitious school with a crowd of people walking around and toward the observer (Figure 1B). The scene was presented in a dynamic continuous fashion and viewed from a fixed, first-person perspective. Participants were instructed to select the target they wanted to search for (the principal of the school) from four possible options balanced for gender and race and viewed independently as they rotated about the y axis. Participants were then instructed to search, locate, and then pursue the target principal walking in a crowded hallway as soon as they appeared from one of eight possible entrances and follow them until no longer visible on the screen. The interval between a target disappearing and reappearing in the hallway from trial to trial varied between 5 and 15 s. The duration of the target’s visibility was primarily determined by its starting point and path length. This varied between 5 and 17 s for the closest and furthest start points respectively.

The primary manipulation of interest was crowd density, which ranged from 1 to 20 people serving as distractors. Crowd density was categorized as low (average of 5 ± 5 people), medium (10 ± 5), and high (15 ± 5), with each level of crowd density presented equally and in pseudo-random fashion. Note that for the purposes of a continuous and free-flowing scene presentation, there was partial overlap in these ranges in part because distractors continuously entered and exited the hallway during an experimental run. In this manner, categorization was determined by the sustained average number of distractors present over the course of a specific trial. For each level of crowd density, participants experienced an equal number of trials for left/right starting points and door distance (but not every possible combination was covered for each level of crowd density). This was done to allow for a simpler factorial design while ensuring that the target path variables did not interfere with the primary manipulation of interest. Each run lasted approximately 3.5 min, and participants completed 3 runs of the experiment with a brief rest period in between. Total testing time was approximately 15 to 20 min for each participant (for further details regarding task design and preliminary results, see [28]).

### 2.4. Behavioral Data Capture, Outcome Measures, and Analysis

Visual search performance was analyzed based on captured eye-tracking data while participants initially searched, located, and then fixated/followed the target. Two primary objective outcomes were collected for this purpose. First, mean success rate (expressed as percent correct responses) was determined based on whether a participant was able to find and fixate on the target in a given trial. Successful fixation criterion was defined as sustained gaze remaining within the outer contour of the target for a minimum time of 0.4 s. Second, mean reaction time (expressed in msec) was defined as the first moment gaze arrived within the outer contour of the target and remained fixated for the duration of the presentation [27,28]. 

Three secondary outcomes were analyzed to further characterize search performance. Gaze error (expressed in arc degrees) was defined as the distance between the center of the target and the participant’s gaze position. This was calculated based on the sampling rate of the eye tracker (90 Hz) and served as a measure of target localizing and fixation accuracy [27,28,33]. Visual search area (expressed as a percent of screen area) was determined based on an ellipse-shaped 95% confidence interval fitted to the captured eye-tracking data. This represented a measure of search precision [27,28]. Finally, we also determined how often/long participants were able to maintain their gaze within the area of the screen based on the continuous recording of eye-gaze coordinate positions. For this purpose, off-screen performance (expressed as a percent) was calculated based on the proportion of gaze points that fell outside of the bounds of the screen on each trial. This outcome served as an index of testing compliance and reliability [27,28,33]. 

Statistical analyses were carried out using SPSS Statistics package version 24 (IBM; Armonk, NY, USA) and JASP (version 0.16.2, https://jasp-stats.org/ (accessed on 23 May 2022)). A non-parametric analysis was pursued following confirmation that recorded data were not normally distributed, and variances were non-homogenous. A series of initial Mann–Whitney *U* tests followed by a Bonferroni correction (adjusted alpha threshold of 0.01) for multiple comparisons was used to investigate between group effects. The effect of task demand (task load) was evaluated using a separate Friedman test for both testing groups. Task differences with respect to success rate and reaction time were determined using a non-parametric repeated-measures Wilcoxon signed-rank test following corrections for multiple comparisons (adjusted alpha threshold of 0.008). Group differences in performance based on visual acuity were evaluated using Mann–Whitney *U* tests following corrections for multiple comparisons (adjusted alpha threshold of 0.0125). Effect sizes were reported as Cohen’s d and partial eta squared for Mann–Whitney *U* tests and Wilcoxon signed-rank tests, respectively. These were calculated using Psychometrica; https://www.psychometrica.de/effect_size.html (accessed on 23 May 2022)). Effect sizes for Friedman tests were reported as Kendall’s coefficient of concordance (Kendall’s W) and calculated using JASP. No data outliers were removed as part of the analysis. 

## 3. Results

### 3.1. Experimental Task 1: Virtual Toybox General Behavioral Results

All the possible toys were selected by both groups, with the yellow duck appearing to be the most commonly chosen target (yellow duck: 54.90% (controls = 55.56%, CVI = 54.17%), orange basketball: 23.53% (controls = 25.93%, CVI = 20.83%), blue truck: 21.57% (controls = 18.52%, CVI = 25.00%)). However, this observed difference in overall distribution frequency was not statistically significant (*Χ*^2^(2, n = 51) = 0.39, *p* = 0.82, d = 0.18). 

**Figure 2:**
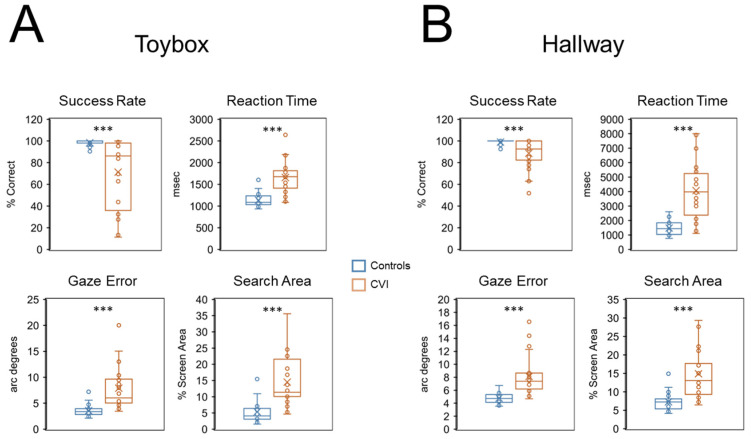
Group comparisons of performance across visual search outcomes for the (**A**) virtual toybox and (**B**) virtual hallway tasks. Comparing group performance revealed that for both tasks, CVI participants were less likely and took longer to find the target as well as had gaze patterns that were less accurate and less precise than controls (as indexed by success rate, reaction time, gaze error, and visual search area, respectively). Results are shown as box plots with interquartile ranges as well as maximum and minimum values (excluding outliers). Individual data (circles) are overlaid with the mean (X) and median value (line) shown. Between group statistical significance levels: *** *p* < 0.001.

In general, CVI participants showed an overall impairment in visual search performance compared to controls with respect to all behavioral outcomes (Figure 2A). For the primary outcomes of interest, mean success rate was significantly lower in the CVI group (71.27% correct ± 30.86 SD) compared to controls (98.41% correct ± 2.42 SD) (U = 557, Z = 4.47, *p* < 0.001, d = 1.56). We also found that mean reaction time was significantly greater in the CVI group (1657.52 msec ± 367.52 SD) compared to the control group (1134.83 msec ± 146.66 SD) (U = 53, Z = 5.11, *p* < 0.001, d = 2.05). These results suggest that CVI participants were less likely and took longer to find the target toy compared to controls.

Regarding secondary outcomes, we found that mean gaze error was significantly higher in CVI (7.90 arc degrees ± 4.04 SD) compared to controls (3.56 arc degrees ± 1.11 SD) (U = 43, Z = 5.30, *p* < 0.001, d = 2.22), indicating that eye-gaze patterns to the target were less accurate in CVI participants. Furthermore, mean search area in the CVI group was significantly greater (14.52% screen area ± 7.52 SD) than in controls (5.36% screen area ± 3.58 SD) (U = 61, Z = 4.96, *p* < 0.001, d = 1.93). This observation suggests that eye movements to the target were less precise in CVI compared to controls. 

Regarding task compliance, we calculated mean off-screen percent values and found a statistically significant difference between the two groups (compare CVI: 3.06% ± 5.06 SD and controls 0.49% ± 0.81 SD) (U = 123.5, Z = 3.78, *p* < 0.001, d = 1.25). The comparatively low values suggest that both groups were able to maintain a high level of task compliance and engagement. However, the significantly higher value in CVI participants suggests that as a group, they were not able to keep their gaze on the screen as well as controls. 

**Figure 3:**
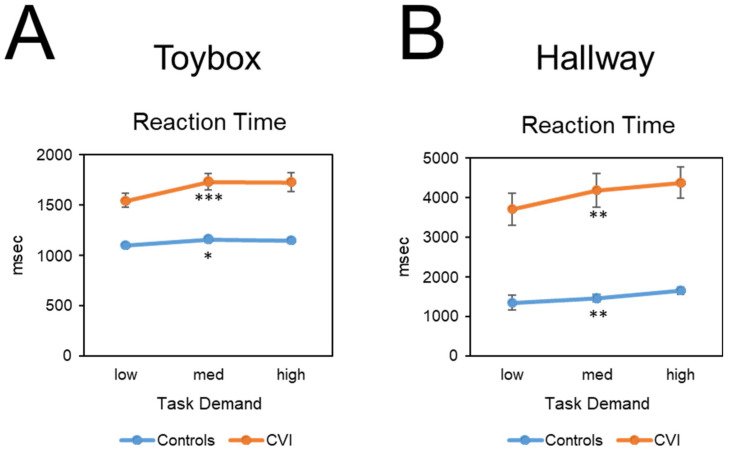
Reaction time performance plotted as a function of task demand for the (**A**) virtual toybox and (**B**) virtual hallway tasks. Overall, reaction times were higher for participants with CVI in each condition and in both tasks. For the virtual toybox, reaction times for both CVI participants and controls were significantly higher on the high compared to low task demand conditions. For the virtual hallway, reaction times in CVI as well as control participants were significantly higher on the high compared to low task demand conditions. Note that, in general, reaction times were also slower for both groups on the virtual hallway compared to toybox task. Error bars represent ± SEM. Individual group load effects statistical significance levels: * *p* < 0.05; ** *p* < 0.01; *** *p* < 0.001.

Friedman tests were conducted to determine whether performance outcomes (reaction time, gaze error, visual search area, and off-screen percent) varied as a function of task demand (i.e., task load). For the CVI group, there was a significant difference in mean reaction time between task demand conditions (*Χ*^2^(2) = 14.08, *p* < 0.001, W = 0.29) (Figure 3A). There was also a significant difference in mean reaction times across task demand conditions for the control group (*Χ*^2^(2) = 8.07, *p* < 0.05, W = 0.15). For both groups, reaction time increased as task demand increased. To compare the magnitude of increase between groups, we conducted a Mann–Whitney *U* test which showed a significant difference in the magnitude of change in reaction times between the low and high task conditions and between the CVI participants (182.47 msec ± 244.10 SD) and controls (50.45 msec ± 115.61 SD) (U = 217, Z = 2.02, *p* < 0.05, d = 0.59). The greater change in mean reaction time in CVI suggests that as a group, they were more affected by increasing task demand. 

For the CVI group, there was a significant difference in mean gaze error across task demand conditions (*Χ*^2^(2) = 12.58, *p* < 0.01, W = 0.26) with a trend for increased gaze error with increased task demand. However, there was no significant difference in mean gaze error across task demand conditions for the control group (*Χ*^2^(2) = 2.30, *p* = 0.32, W = 0.04). This suggests that in CVI, visual search accuracy significantly worsened with increasing task demand. 

For the CVI group, there was a significant difference in visual search area across task demand conditions (*Χ*^2^(2) = 12.58, *p* < 0.01, W = 0.26). There was also a significant difference in visual search area across task demand conditions for the control group (*Χ*^2^(2) = 12.52, *p* < 0.01, W = 0.23). Thus, there was a trend of increased visual search area with increasing task demand in both groups. As a post hoc analysis, we conducted a Mann–Whitney *U* test, which revealed that there was no significant difference in the magnitude of change for visual search area across task demand between the CVI group (1.35% screen area ± 2.80 SD) and controls (0.86% screen area ± 1.09 SD) (U = 287, Z = 0.70, *p* = 0.49, d = 0.20). 

Finally, there was no significant difference in off-screen percent values across task demand conditions for either the CVI participants (*Χ*^2^(2) = 0.26, *p* = 0.88, W = 0.01) and controls (*Χ*^2^(2) = 0.82, *p* = 0.664, W = 0.02). These findings indicate similar levels of task compliance across difficulty levels for both groups.

### 3.2. Experimental Task 2: Virtual Hallway General Behavioral Results

All the possible targets were selected by both groups, with the exception of the Black female principal, which was only selected by the control group. Overall, the White female appeared as the most commonly chosen target (White female: 61.22% (controls = 56.00%, CVI = 66.67%), White male: 22.45% (controls = 20.00%, CVI = 25.00%), Black female: 10.20% (controls = 20.00%, CVI = 0.00%), Black male: 6.12% (controls 4.00%, CVI = 8.33%)). However, the observed difference in overall distribution frequency was not statistically significant (*Χ*^2^(3, n = 49) = 5.54, *p* = 0.136, d = 0.71). 

As with the toybox task, participants in the CVI group showed an overall impairment in visual search performance compared to controls with the virtual hallway task (Figure 2B). Mean success rate for the CVI group (88.72% correct ± 13.66 SD) was significantly lower compared to controls (98.96% correct ± 2.27 SD) (U = 476.50, Z = 3.87, *p* < 0.001, d = 1.17). We also found that mean reaction time was significantly greater in the CVI (4071.02 msec ± 1913.43 SD) compared to the control group (1483.60 msec ± 460.90 SD) (U = 48, Z = 5.04, *p* < 0.001, d = 2.08). Similar to the findings with the toybox, these results suggest that CVI participants were less likely and took longer to find the target principal compared to controls.

Regarding secondary outcomes, we found that mean gaze error was significantly higher in CVI (8.12 arc degrees ± 2.98 SD) compared to controls (4.79 arc degrees ± 0.78 SD) (U = 34, Z = 5.32, *p* < 0.001, d = 2.34). Furthermore, mean visual search area in the CVI group (14.87% screen area ± 6.67 SD) was significantly greater than in controls (7.30% screen area ± 2.40 SD) (U = 58, Z = 4.84, *p* < 0.001, d = 1.91). Again, similar to the findings with the toybox, these results suggests that eye-gaze patterns to the target were less accurate and less precise in CVI participants compared to controls.

For task compliance, we calculated mean off-screen percent values and found no statistically significant difference between the two groups (compare CVI: 0.92% ± 1.73 SD, and controls 0.35% ± 0.31 SD) (U = 245.50, Z = 1.09, *p* = 0.28, d = 0.36). The comparatively low and comparable off-screen percent values are indicative of both groups maintaining a relatively high level of compliance and engagement on this task.

Friedman tests were conducted to determine whether the performance outcomes of reaction time, gaze error, visual search area, and off-screen percent varied significantly as a function of task demand (i.e., task load). For the CVI group, there was a significant difference in mean reaction time across task demand conditions (*Χ*^2^(2) = 10.58, *p* < 0.01, W = 0.22) (Figure 3B), with reaction time increasing with increasing task demand. There was also a significant difference in mean reaction time across task demand conditions for the control group (*Χ*^2^(2) = 10.64, *p* < 0.01, W = 0.21), with reaction time increasing with increasing task demand. A Mann–Whitney *U* post hoc test revealed no significant difference in the magnitude of change with respect to reaction time between the CVI (668.86 msec ± 991.78 SD) and control groups (306.45 msec ± 864.62 SD) (U = 284, Z = 0.32, *p* = 0.76, d = 0.09). 

For the CVI group, there was no significant difference in mean gaze error between task demand conditions (*Χ*^2^(2) = 5.08, *p* < 0.08, W = 0.12). There was, however, a significant difference in mean gaze error across task demand conditions for the control group (*Χ*^2^(2) = 17.36, *p* < 0.001, W = 0.35), with increasing gaze error with increased task demand. This indicates that task demand significantly affected gaze error for controls but not for the CVI group. 

There was no significant difference in visual search area for the CVI group across task demand conditions (*Χ*^2^(2) = 4.08, *p* = 0.13, W = 0.09). There was, however, a significant difference with respect to visual search area for controls (*Χ*^2^(2) = 10.64, *p* < 0.01, W = 0.21), with a trend of increasing visual search area with increasing task demand. This indicates that task demand significantly affected visual search area for controls but not for the CVI group.

Finally, there was no significant difference in off-screen percent values in the CVI (*Χ*^2^(2) = 5.70, *p* = 0.06, W = 0.12) and for the control group (*Χ*^2^(2) = 0.02, *p* = 0.99, W = 4.21 × 10^−4^) across task demand conditions. These findings indicate similar levels of task compliance and engagement for both groups. 

### 3.3. Comparison of Performance between Tasks

To investigate whether the CVI and control groups performed differently on the virtual toybox and hallway tasks, we conducted Wilcoxon signed-rank tests with respect to success rate, reaction time, and off-screen percent values. (Note that the stimulus dimensions were not the same for both tasks, and therefore, we did not compare gaze error and visual search area outcomes.) For the CVI group, there was a significant difference in success rate between the toybox (71.27% correct ± 30.86 SD) and the hallway task (88.72% correct ± 30.86 SD) (W = 219, Z = 3.00, *p* < 0.01, η^2^ = 4.46). The CVI group had a significantly lower success rate in the toybox compared to the hallway, suggesting that CVI participants were better at finding and pursuing the target in the hallway task. For the control group, there was no significant difference in success rate between the toybox (98.41% correct ± 2.42 SD) and hallway task (98.96% correct ± 2.27 SD) (W = 42, Z = 0.80, *p* = 0.45, η^2^ = 10.32).

For the CVI group, there was a significant difference in mean reaction time between the toybox (1657.52 msec ± 367.52 SD) and hallway task (4071.02 msec ± 1913.43 SD) (W = 273, Z = 4.12, *p* < 0.001, η^2^ = 3.13). For the control group, there was also a significant difference in reaction time between the toybox (1134.83 msec ± 146.66 SD) and hallway task (1483.60 msec ± 480.59 SD) (W = 212, Z = 2.78, *p* < 0.01, η^2^ = 4.14). The significantly lower reaction times for the toybox compared to the hallway task indicate that both groups were faster at finding the target in the toybox task.

Regarding task compliance, for the CVI group, there was a significant difference in off-screen percent between the toybox (3.06% ± 5.12 SD) and hallway task (0.96% ± 1.77 SD) (W = 30, Z = 3.29, *p* < 0.001, η^2^ = 10.96). The significantly greater off-screen percent in the toybox compared to the hallway task indicates that CVI participants maintained better task compliance in the hallway task. For the control group, there was no difference in off-screen percent between the toybox (0.96% ± 1.77 SD) and hallway task (0.38% ± 0.31 SD) (W = 136, Z = 0.71, *p* =0.49, η^2^ = 6.56). 

#### Effect of Visual Acuity on Task Performance 

**Figure 4:**
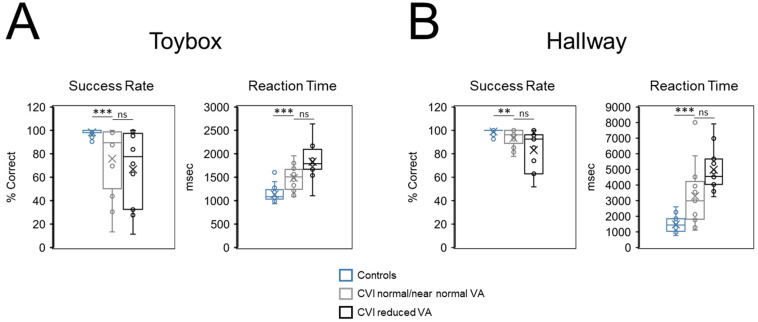
Comparison of visual search performance in CVI participants separated by normal/near normal (20/15 to 20/25 Snellen) to reduced (20/30 to 20/100) visual acuity. (**A**) In the toybox task, CVI participants with normal/near normal visual acuity showed a significantly lower success rate and longer reaction times when compared to controls. Performance was not significantly different between CVI participants with normal/near normal and impaired visual acuities with respect to success rate. Similarly, reaction times were not significantly slower in the impaired visual acuity CVI group. (**B**) In the hallway task, CVI participants with normal/near normal visual acuity showed a similar pattern of significantly lower success rate and longer reaction times compared to controls. Performance was not statistically significant between CVI participants with normal/near normal and impaired visual acuities with respect to success rate. Similarly, reaction times were not significantly slower in the impaired visual acuity CVI group. Results are shown as box plots with interquartile ranges as well as maximum and minimum values (excluding outliers). Individual data (circles) are overlaid with the mean (X) and median value (line) shown. Between group statistical significance levels: ** *p* < 0.01; *** *p* < 0.001; n.s., not significant.

CVI participants were categorized as having either reduced (equal or worse than 20/30 Snellen; n = 12) or normal/near normal (equal or better than 20/25 Snellen; n = 12) visual acuity. For the toybox task, there was no significant effect of visual acuity on success rate between the reduced (66.67% ± 32.31 SD) and normal/near normal visual acuity groups (75.87% ± 30.02 SD) (U = 88.5, Z = 0.95, *p* = 0.35, d = 0.40) (Figure 4A). There was a trend for longer reaction time in the reduced acuity CVI group (1831.75 msec ± 378.93 SD compared to the normal/near normal acuity group (1483.30 msec ± 269.90 SD). However, after correcting for multiple comparisons, this trend was not statistically significant (U = 35, Z = 2.14, *p* = 0.03, d = 0.97). When compared to controls, CVI participants with normal/near normal visual acuity showed significantly lower success rates (U = 53.50, Z = 3.41 *p* < 0.001, d = 1.201) and longer overall reaction times (U = 291, Z = 3.93, *p* < 0.001, d = 1.62) (Figure 4A).

The same comparison analysis was carried out for the virtual hallway task for CVI participants with reduced (equal or worse than 20/30 Snellen; *n* = 11) and normal/near normal (equal or better than 20/25 Snellen; *n* = 13) visual acuity. There was no significant effect with respect to success rate between the reduced acuity (83.14% ± 17.22 SD) and normal/near normal acuity CVI groups (93.45% ± 7.58 SD) (U = 97, Z = 1.50, *p* = 0.15, d = 0.63) (Figure 4B). There was a trend for greater reaction time in the reduced acuity CVI group (4985.00 msec ± 1423.54 SD) compared to the normal/near normal acuity group (3297.66 msec ± 1978.63 SD). However, after correcting for multiple comparisons, this trend was not statistically significant (U = 30, Z = 2.40, *p* < 0.02 d = 1.13). When compared to controls, CVI participants with normal visual acuity showed significantly lower success rates (U = 86, Z = 2.78, *p* < 0.01, d = 0.83) and significantly longer overall reaction times than controls (U = 277, Z = 3.52, *p* < 0.001, d = 1.39) (Figure 4B).

## 4. Discussion

We assessed visual search performance while study participants interacted with two novel VR-based naturalistic environments as a means to objectively characterize functional vision abilities. The results obtained from this study are consistent with the prominence of higher-order visual processing deficits observed in CVI [9,12] and reports of how individuals with CVI face challenges with selecting and extracting visual information from complex scenes [12,13,14,15]. 

Using our virtual toybox and hallway tasks, we found that CVI participants showed an overall impairment in search performance on all of our outcomes of interest when compared to chronologically age-matched controls. In general, CVI participants were less likely and took longer to find the target (as indexed by decreased mean success rates and increased reaction times, respectively). Further, eye-gaze patterns were less accurate and less precise (as indexed by increased measures of gaze error and visual search area, respectively). Consistent with previous studies, we also observed that behavioral responses within the CVI group showed a wider range of inter-individual variability compared to control participants (e.g., [34,35]). Task engagement and compliance on both our tasks was generally high and comparable between CVI participants and controls, as indexed by the off-screen percentage metric. Specifically, off-screen percent values were not significantly different between CVI participants and controls for the hallway task. However, they were significantly higher in the CVI group for the toybox, suggesting that their testing compliance was lower for this task. 

We also found that CVI participants showed a trend of worsening performance with increasing task demand on both our VR-based tasks. Specifically, we found that reaction times increased with increasing clutter (i.e., number of unique distractor toys in the toybox task) and with increasing crowd density (i.e., number of distractor individuals in the hallway task). This trend of worsening performance with increasing task demand was also observed with gaze error and visual search area values, which were consistent with less accurate and precise gaze patterns. When comparing performance across both tasks, we found that CVI participants were more likely to find the target on the hallway task, but they were faster in finding the target (i.e., lower reaction time) for the toybox task. 

These group discrepancies regarding performance are likely related to differences in the design of the two VR-based environments. It is possible that the highly dynamic and continuously flowing visual stimulus associated with the hallway task was more engaging and/or attentionally demanding for CVI participants. Thus, while they were more likely to find the target and maintain their gaze (i.e., pursuit eye movements) on the screen, they were nonetheless slower in finding the target compared to the toybox task. In contrast, the static nature and sequential presentation of trials associated with the toybox task may have allowed CVI participants to find the target more quickly. However, sustaining fixation on the static target may have been more challenging. Furthermore, there are also key differences in terms of the duration of target presentation between the two tasks that also limit comparisons that can be drawn with respect to overall performance. In the toybox, the target was presented in a serial fashion and was always present for the entire length of the trial. In contrast, the target was not always present on the screen for the hallway (thus requiring constant search behavior), and target presentation time varied between 5 to 15 s depending on the path taken. These design differences may have influenced a CVI participant’s ability to search, locate, and fix/pursue the target. This could help explain observed differences with respect to success rate and reaction time results across the two tasks. 

Importantly, these differences between control and CVI participants more likely reflect underlying neurophysiology rather than impaired entering levels of visual function or overall poor testing compliance. In fact, all CVI participants had intact visual field function within the area corresponding to testing, and there was evidence of impaired search performance in CVI participants having visual acuities at normal/near normal levels. Specifically, success rates and reaction times on both the toybox and hallway tasks were comparable when CVI participants were separated based on their visual acuities. Crucially, these same performance outcomes in CVI participants with normal/near normal visual acuity were significantly worse on both tasks when compared to controls. 

Our finding of visual search deficits in CVI in the absence of impaired visual acuity is in line with a number of reports assessing different aspects of visual processing. For example, Kooiker and colleagues demonstrated clear impairments in children with CVI with respect to visual search as well as fixation and ocular motor pursuit [36,37,38]. Another study by our group showed that compared to controls, CVI participants had a significantly higher mean motion-coherence threshold (determined using a random dot kinematogram pattern simulating optic flow motion) despite having visual acuities within normal range [35]. These global motion processing deficits in CVI appeared to be associated with impaired signal integration and segregation mechanisms at the level of extra striate visual processing areas (i.e., area hMT+), as revealed by functional magnetic resonance imaging (fMRI) [35]. A recent report by Chandna and colleagues (2021) tested steady-state visual evoked potentials (SSVEPs) in response to visual motion in 31 children with CVI good binocular visual acuity (mean of 0.12 ± 0.11 SD logMAR). Compared to age-matched controls (mean visual acuity of 0.14 ± 0.16 SD logMAR), the authors found that CVI subjects showed significant deficits in the processing of complex (but not elementary) motion patterns [29]. Consistent with the results reported here, the finding of focused higher-order processing deficits further points to the contribution of developmental damage and the maldevelopment of key central visual processing areas. Furthermore, these findings demonstrate the importance of characterizing higher-order visual perceptual difficulties in this population that are not typically assessed as part of a standard ophthalmic examination and therefore can be underestimated or missed [2,22]. 

Simply put, visual search can be described as a process that allows an individual to find and distinguish a target in the environment while ignoring irrelevant surrounding information [26,39]. It is nonetheless a complex behavior that requires not only coordinated movement of the eyes but also neural signaling within a large network of brain areas that integrate numerous sensory, perceptual, and cognitive processes [40]. Key components include identification and visuospatial processing for the encoding of visual properties that distinguish relevant from irrelevant information, along with the deployment of selective attention to a target while ignoring surrounding distractors [41]. The neurophysiological basis of impaired visual search in CVI remains unclear. Current evidence suggests that neurodevelopmental damage is associated with impaired visuospatial processing [42]. Within this context, the presence of higher-order perceptual deficits could certainly be expected in the setting of early neurological injury. Our results appear consistent with the notion that individuals with CVI may have greater difficulty identifying the target, shifting and maintaining their attention, and ignoring distracting stimuli in a complex scene. Taken together, a number of studies have pointed to a likely combination of factors impacting visual search in CVI, including an impaired ability to discriminate a target from surrounding distractors, reduced attentional control processes, as well as impaired ocular motor responses once the target is detected, fixated, and followed [37,38,43,44,45]. 

The two-stream hypothesis (i.e., for dorsal/spatial and ventral/object processing) has often been purported to describe the division of labor regarding how certain attributes of a visual scene are analyzed [46]. In this view, the predominance of higher-order perceptual deficits, including visuospatial and complex motion processing, sensitivity to visual scene complexity, as well as attention deployment and the suppression of distractor elements [47], have led to the proposition that CVI represents a condition characterized by dorsal stream “dysfunction” or “vulnerability”. This would be consistent with the maldevelopment or impaired functioning of the dorsal/spatial processing pathway (i.e., connecting the occipital to parietal cortices and terminating in frontal areas) [48,49,50]. However, many individuals with CVI also exhibit a broad spectrum of visual dysfunctions related to object identification, such as recognizing faces and shapes [4,51,52,53]. In this view, these latter impairments would be consistent with maldevelopment along the ventral processing pathway (i.e., connecting the occipital and temporal cortices). Certainly, both our VR-based tasks tap into functions typically associated with both visual-processing streams. Thus, characterizing our virtual toybox (i.e., static object) and hallway (i.e., dynamic human) tasks as predominantly investigating ventral and dorsal processing abilities, respectively, would represent a gross oversimplification. 

The development of VR-based simulations of naturalistic scenes and behaviorally relevant tasks combined with eye tracking provides an opportunity to characterize functional vision performance in a manner beyond what is typically assessed in a standard ophthalmic exam. The approach offers experimental control so that the effect of manipulating task demands as well as various features of a visual scene can be systematically investigated [16,24]. As a next step, these same VR-based environments can be used to explore environmental adaptations that may be helpful in improving search behaviors on an individual level. These observations could potentially be transferred to corresponding real-world situations. For example, our results demonstrate that individuals with CVI are sensitive to visual task demands (i.e., load) associated with scene complexity. Thus, decreasing visual clutter and crowd density may be helpful in improving search and overall functional vision performance [54,55]. This can be achieved by reducing the number of items in the environment that need to be discriminated, especially if they are of the same shape and color or are overlapping with one another. At the same time, increasing target saliency may also promote identification as well as guide and sustain attention. This could be achieved by altering various target features such as its color, luminance, and motion pattern [56]. Finally, the impact of combining other sensory factors (such as ambient noise levels) can also be explored. Future studies are now ongoing to investigate the effect of these possible manipulations more systematically using these same VR-based environments as well as environmental adaptations that could be tailored at the individual level. 

A number of recent studies are also developing novel approaches using a combination of eye tracking and/or more engaging behavioral tasks to characterize functional vision performance in CVI and early brain injury. Mooney and colleagues (2021) recently developed series of tasks called the “visual ladder” that records and grades gaze-based tracking behavior with respect to spontaneous saccades and pursuits, visual field sensitivity, and spatial visual functions [57]. Responses from 10 non-communicative children with CVI (aged from 3 to 18 years) and with diverse causes of brain injury were recorded to support the viability of this novel assessment approach. Results were useful in generating an individualized profile of important visual functions, including biases in eye movement direction, ocular motor abnormalities (e.g., nystagmus), as well as visual field and contrast sensitivity impairments [57]. Chang and Borchert (2020) developed a passive viewing experimental protocol combined with eye tracking to assess ocular motor behavior in younger children with CVI with more severe physical and cognitive impairments. Preliminary analysis of results from the free viewing behavior of naturalistic images revealed that children with CVI showed a longer latency initiating saccades while searching for a target in the visual scene [58] (see also [59] using eye tracking to assess visual acuity). Finally, the Austin Playing Card Assessment developed by McDowell (2020) represents a novel screening method to help identify visual perceptual difficulties in CVI [20]. Using standard playing cards, a participant is instructed to pick up pairs of cards, while task demand is increased by varying the number of cards in the search array. Using this simple approach, the author found that individuals with CVI were slower in finding target pairs compared to controls. Furthermore (and consistent with the observations reported in our toybox task), performance worsened with increasing task demand. Future studies are looking to digitize the Austin Playing Card Assessment testing platform and incorporate eye-tracking metrics [20]. 

Considering the inclusion/exclusion criteria of this study, it is evident that CVI participants required a certain level of visual acuity and ocular motor function to permit accurate eye-tracking calibration and data capture. This issue is likely to limit the generalizability of our observed results, especially when considering the broad and heterogeneous clinical profile associated with this condition. Thus, to avoid this potential selection bias, it is important that future studies employ VR-based designs that are adapted to accommodate participants with an even wider range of visual acuities, oculomotor functions, and cognitive abilities. Furthermore, future studies should also incorporate a broader capture of standardized neuropsychological metrics (such as verbal IQ) to allow for group matching based on developmental abilities as well as systematic characterization of underlying neurological damage. The collection of this information could be helpful in revealing potential associations between these factors and visual search and functional vision performance. 

## 5. Conclusions

In this study, we sought to characterize higher-order visual processing abilities in CVI in relation to visual search performance. We found that CVI participants showed impaired search abilities in both of our VR-based tasks. Furthermore, they were sensitive to task demands related to the complexity of the visual scene. Crucially, these observed impairments with searching could not be solely explained by reduced visual function or poor testing compliance on the part of our CVI participants. The value of this combined VR-based and eye-tracking approach may help to further characterize the nature of visual perceptual deficits in CVI and potentially explore helpful environmental modifications and adaptive strategies on an individual basis as well as group level.

## Data Availability

The datasets generated during and/or analyzed during the current study are available from the corresponding author upon reasonable request and IRB approval.
